# Identification of D/Yama2019 Lineage-Like Influenza D Virus in Chinese Cattle

**DOI:** 10.3389/fvets.2022.939456

**Published:** 2022-07-14

**Authors:** Jieshi Yu, Tianyu Li, Zhenyu Wen, Siyu Wu, Zhilin Wang, Jiaying Zheng, Mingwang Chen, Faming Chen, Wen-Kang Wei, Shao-Lun Zhai, Ming Liao

**Affiliations:** ^1^Agro-biological Gene Research Center, Guangdong Academy of Agricultural Sciences, Guangzhou, China; ^2^Key Laboratory for Prevention and Control of Avian Influenza and Other Major Poultry Diseases, Ministry of Agriculture and Rural Affairs, Scientific Observation and Experiment Station of Veterinary Drugs and Diagnostic Techniques of Guangdong Province, Ministry of Agriculture and Rural Affairs, Key Laboratory of Livestock Disease Prevention of Guangdong Province, Institute of Animal Health, Guangdong Academy of Agricultural Sciences, Guangzhou, China; ^3^College of Animal Science and Technology, Guangxi University, Nanning, China; ^4^Zhongshan Animal Disease Control Center, Zhongshan, China; ^5^Key Laboratory of Zoonosis of Ministry of Agriculture and Rural Affairs, Key Laboratory of Zoonosis Prevention and Control of Guangdong Province, South China Agricultural University, Guangzhou, China; ^6^Maoming Branch Center of Guangdong Laboratory for Lingnan Modern Agricultural Science and Technology, Maoming, China

**Keywords:** influenza D virus, genetic lineage, phylogenetic analysis, cattle, China

## Abstract

Outbreaks of influenza D virus (IDV) continue to be reported in many countries. On the basis of the hemagglutinin-esterase fusion (HEF) gene, five IDV genetic lineages have been identified: D/OK, D/660, D/Yama2016, D/Yama2019 and D/CA2019 lineages. Previously reported IDV strains in China all form a sub-clade (D/China sub-lineage) within D/OK lineage. From October 2021 to February 2022, nasal swab samples (*n* = 250) were collected from apparently healthy cattle in slaughterhouses around the city of Guangzhou, China, and screened for IDV by RT-PCR. Ten samples were positive for IDV. An IDV strain with nearly complete genome sequences was identified and designated as D/bovine/CHN/JY3001/2021. Importantly, sequence alignments and phylogenetic analyses revealed that this IDV strain is genetically close to the strains (>98% homology) in the D/Yama2019 lineage that has been found only in Japan, but distant from the previously reported Chinese IDV strains (~95% similarity). These results demonstrate the emergence of D/Yama2019 lineage IDV in Chinese cattle herds, highlighting a need for future surveillance of D/Yama2019-like viruses toward better understanding both epidemiology and diversity of IDV in China.

## Introduction

Influenza D virus (IDV) was first reported in 2011 and has been found in a wide range of animal species all over the world (1, 2). Unlike influenza A, B and C viruses, cattle are the major reservoir of IDV (3). Increasing studies have shown that IDV can be highly associated with bovine respiratory disease (RBD) complex (1, 4) that causes huge economic losses in cattle industry.

Influenza D viruses (IDVs) can be divided into five genetically distinct lineages (D/OK, D/660, D/Yama2016, D/Yama2019 and D/CA2019) based on the hemagglutinin-esterase fusion (HEF) gene (5). D/OK lineage is so far the most frequently reported lineage worldwide, while D/660 lineage is mainly distributed in Europe and North America. Interestingly, both D/Yama2016 and D/Yama2019 lineages seem to be restricted in Japan (1, 6–8). Recently, a novel phylogenetic lineage of IDV with broad antigenicity has been discovered in California, USA, which has been named D/CA2019 (5).

The first-ever detection of IDV was documented in China in 2014, and three strains of bovine IDVs were identified in Shandong, northern China (9). In 2016-2017, a study investigating the prevalence of IDV in southern China found that IDVs were widely present in different farm animal species including dairy cattle, yellow cattle, buffalo, pigs and goats, and the positive rates varied from 4 to 40% (10). Besides southern and northern China, six IDV positive samples were recently detected from swine in Eastern China (11). The results from these studies indicated that IDVs may be prevalent throughout China. Phylogenetic analyses of IDVs in China suggested that they formed a distinct sub-clade (D/China sub-lineage), but all these isolates belong to D/OK lineage (11).

The continued circulation of IDV in Chinese cattle and swine herds, combined with its potential for causing respiratory illness outbreaks in cattle, prompts further investigation of epidemic characteristics and evolutionary dynamics of IDV in China. Here, we describe the first detection of D/Yama2019 lineage-like IDV, and genetically characterize this emerging IDV toward better understanding of the genetic diversity of IDVs in China.

## Materials and Methods

### Sampling, PCR Detection and Sequencing

**Figure 1:**
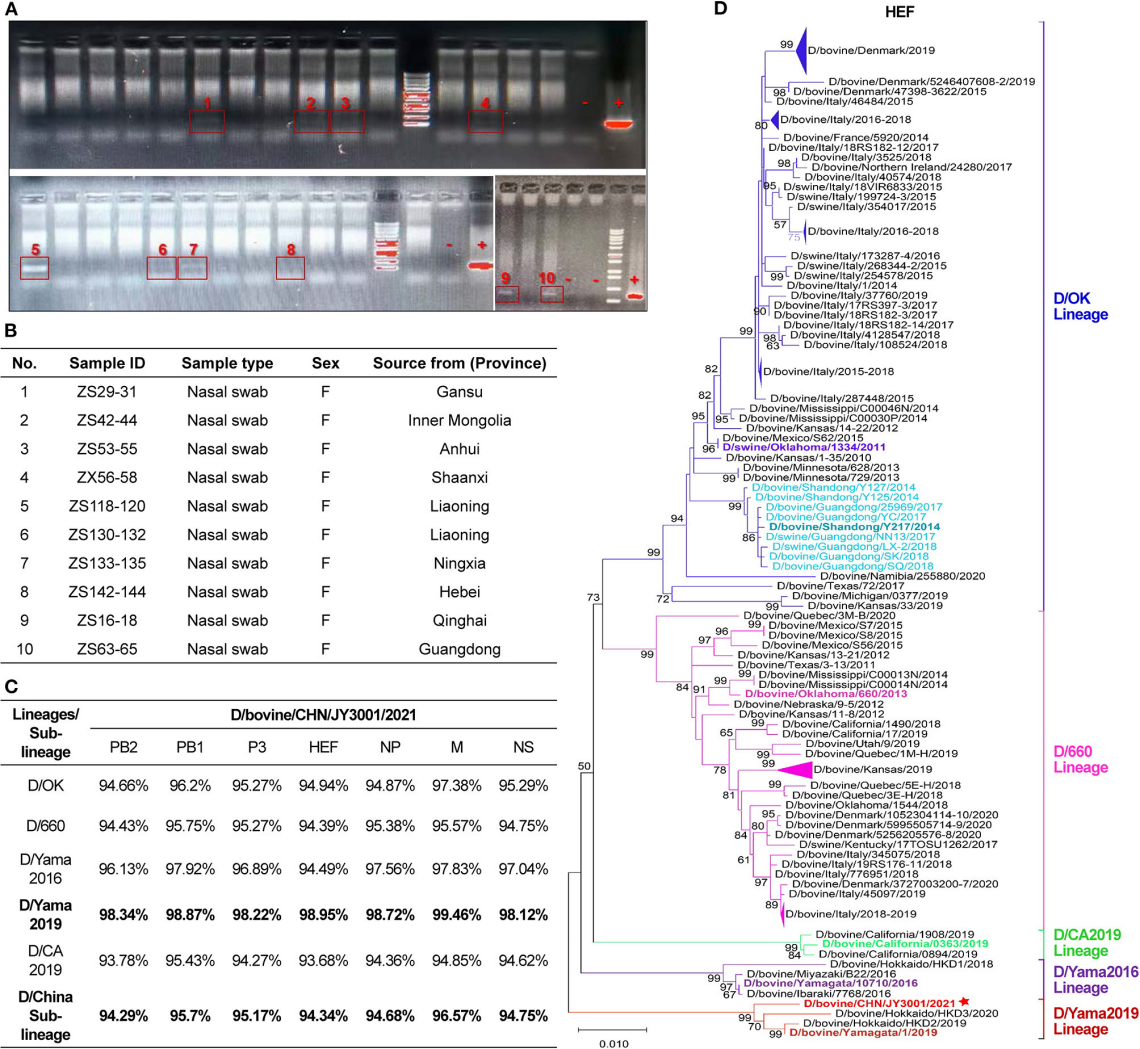
Detection and characterization of influenza D virus in nasal swab samples of cattle. **(A)** Agarose gel electrophoresis analysis of the amplicon products resulting from RT-PCR using the designated forward and reverse primers to amplify a fragment of 382 bp of IDV P42 gene. IDV-positive samples were labeled and highlighted with red boxes. “–”: negative controls; “+”: positive controls. **(B)** IDV-positive samples identified in this study. The sample ID and type reported together with animal information (sex and source). **(C)** Nucleotide sequence alignments of individual segments of the IDV isolate found in this study and representative strains of the D/OK lineage, D/660 lineage, D/Yama2016 lineage, D/Yama2019 lineage, D/CA2019 lineage and D/China sub-lineage. **(D)** Maximum-likelihood phylogenetic tree of D/bovine/CHN/JY3001/2021 and other known IDV strains. Nucleotide sequences of HEF genetic segment were aligned and analyzed using MEGA- X, with 1,000 bootstrap replicates. Bootstrap scores of at least 50 were shown to the left of the major nodes. Scale bar represents the number of substitutions per site. Each branch of five present lineages of IDV (D/OK-, D/660-, D/CA2019-, D/Yama2016-, and D/Yama2019-lineage) and the D/China sub-lineage was noted by blue, pink, green, purple, red and light blue color, respectively. The branch of D/bovine/CHN/JY3001/2021 was marked with a star. The D/bovine/CHN/JY3001/2021 and representative strains described in this study are bolded. Triangles indicate compressed branches with virus strains isolated in the same locations.

During the winter and spring of 2021/2022, 250 nasal swab samples from apparently healthy cattle were collected in slaughterhouses around the city of Guangzhou, China. The cattle were sourced from various parts of the country including Gansu, Inner Mongolia, Anhui, Guangdong, Shaanxi, Liaoning, Ningxia, Hebei and Qinghai provinces. Viral RNA was extracted from above samples by using the Kit (Omega, E.Z.N.A.^®^ Viral RNA Kit) and the isolated RNA was converted to cDNA by using the reverse transcription kit (Vazyme, HiScript^®^ III 1st Strand cDNA Synthesis Kit), according to the manufacturer's instructions. For IDV detection, PCR assays with a pair of primers targeting P42 gene were conducted to generate an amplicon of 382bp. The forward primer is IDV-M-364-F: 5′- GATGTATGAAATGAGGGAGGAC-3′, and the reverse primer is IDV-M-746-R: 5′-AAGATTAGCCATTCCACTGAC-3′ (Table 1). The sample with the most intense PCR band (Figure 1A) was selected for further molecular characterization. Reverse transcription PCR (RT-PCR) of all seven genome segments of the isolate (D/bovine/CHN/JY3001/2021) was performed using conserved IDV-specific primers (Table 1). RT-PCR products were purified *via* an agarose gel DNA extraction kit (Omega, Gel Extraction Kit) and then Sanger sequenced with the IDV-specific primers. The sequences were assembled and proofread manually. The sequences were deposited in GenBank under accession numbers (ON415263-ON415269).

**Table 1:** Primers used in this study.

**No**.	**Primer name**	**Sequence (5^′^-3^′^)**	**No**.	**Primer name**	**Sequence (5^′^-3^′^)**
1	IDV-M-364-F	GATGTATGAAATGAGGGAGGAC	14	IDV-P3-2134-R	CTCACTCAAAGTATTTAGCTCCATTGGA
2	IDV-M-746-R	AAGATTAGCCATTCCACTGAC	15	IDV-HEF-25-F	ATGTTTTTGCTTCTAGCAACAATTACAG
3	IDV-PB2-17-F	TGTCACTACTATTAACGCTCGCAAAAG	16	IDV-HEF-1104-R	GAAAATACATCCTTCTGTTTGTAGGC
4	IDV-PB2-1045-F	GAAAGTCAGTGGAGAAGCAGAAACA	17	IDV-HEF-982-F	TCATATTGCTTCGACACTGATGGAG
5	IDV-PB2-1284-R	GATTCAAAGAATCTCCCATCTCTGCA	18	IDV-HEF-2023-R	GATTCTATTTCTTGCAACAGATCCA
6	IDV-PB2-2335-R	TCAAACTTCCAGACGCATTCTACGAA	19	IDV-NP-4-F	GACTCAACAAAAGTCCAAACGCCT
7	IDV-PB1-1-F	ATGGAAATAAACCCATATCTACTCATG	20	IDV-NP-816-F	GATAATGCCTTGGTCAATGTGGCT
8	IDV-PB1-996-F	CAACTTAATGAAAGATCTCTGCTCAG	21	IDV-NP-1114-R	TCAACTGATATCTTTTCTTCATGGGTTG
9	IDV-PB1-1243-R	GTTAAACATTCCCATCAGCATTCCT	22	IDV-NP-1648-R	CAACTGTTTCAACGTCCATACTTGA
10	IDV-PB1-2244-R	ACGCTTGGCGTCTTCGATTAGT	23	IDV-M-13-F	CAACTACTTGCTGAACTTGAGGGAT
11	IDV-P3-1-F	ATGTCTAGTATAATCAGAGAAATCGCAAAG	24	IDV-M-1137-R	TTCATTGAATCCACTCAATGGAGG
12	IDV-P3-867-F	TGATTTCTTTGAAGCAGCAAACATGG	25	IDV-NS-F	GTCTGAAAACAAGTCAGTGAACACA
13	IDV-P3-1229-R	GGCATTTCTGCCATGGGTATCCA	26	IDV-NS-R	TGAAATTGTTCTCGAAACTGACTTG

### Sequence Alignment Analysis

Comparison of the HEF sequences of the D/bovine/CHN/JY3001/2021 isolate and representative strains of the D/OK lineage (D/swine/Oklahoma/1334/2011), D/660 lineage (D/bovine/Oklahoma/660/2013), D/Yama2016 lineage (D/bovine/Yamagata/10710/2016), D/Yama2019 lineage (D/bovine/Yamagata/1/2019), D/CA2019 (D/bovine/California/0363/2019) and D/China sub-lineage (D/bovine/Shandong/Y217/2014) were conducted using Blast tool in NCBI (http://ncbi.nlm.nih.gov).

### Phylogenetic Analysis

IDV genome sequences were downloaded from the GenBank database (NCBI: http://ncbi.nlm.nih.gov) (accessed on April 24, 2022) for phylogenetic analysis. The sequences were aligned with the D/bovine/CHN/JY3001/2021 sequences by ClustalW in MEGA-X (12). Partial gene sequences (<80% of the full-length gene sequences) were not included in the analyses. Phylogenetic trees for individual genome segments were generated using the maximum likelihood method and Tamura-Nei model with 1,000 bootstrap replicates in MEGA-X (12). Branches with <50% bootstrap support were omitted.

### Virus Isolation

The supernatants of the IDV-positive samples were inoculated to Madin-Darby canine kidney (MDCK) cells, Swine testicle (ST) cells and Human Rectal Tumor (HRT-18G) cells on 6-well plates. The plates were incubated at 33°C for 4–5 days. The supernatants were blindly passaged in ST cells. The infectivity was checked by determining hemagglutination (HA) titer or doing RT-PCR. Unfortunately, no IDV was obtained from the virus isolation attempts.

## Results

### RT-PCR Detection, Genome Sequencing and Alignment Analysis

The nasal swab samples (*n* = 250) from apparently healthy cattle were tested for IDV by RT-PCR. Ten clinical samples (4%) were found positive by amplification of partial sequences of P42 gene (Figure 1A). These positive samples were collected from cattle from farms located at different provinces of China (Figure 1B). Only one of the IDV-positive samples with the most-intense PCR band (Figure 1A) could be amplified for individual genomic segments. These PCR products of IDV genomic segments were purified and sequenced. Sequences were assembled and proofread, and then submitted to GenBank (D/bovine/CHN/JY3001/2021, accession number: ON415263-ON415269).

Alignment analyses showed that sequences of the IDV isolate found in this study shared the highest nucleotide similarity (>98%) to that of the representative strain (D/bovine/Yamagata/1/2019) of D/Yama2019 lineage but displayed the lowest nucleotide similarity (<95%) to that of the representative strain (D/bovine/California/0363/2019) of D/CA2019 lineage (Figure 1C). The sequence similarity between the D/bovine/CHN/JY3001/2021 and representative strains of other IDV lineages (D/OK lineage: D/swine/Oklahoma/1334/2011; D/660 lineage: D/bovine/Oklahoma/660/2013; D/Yama2016 lineage: D/bovine/Yamagata/10710/2016) ranged from 94 to 98% (Figure 1C). Moreover, the D/bovine/CHN/JY3001/2021 strain shared relatively low sequence homology (94–95%) with the representative strain of D/China sub-lineage (Figure 1C). These alignment analysis results highlighted a distinct IDV strain circulating in Chinese cattle herds.

### Phylogenetic Analysis

**Figure 2:**
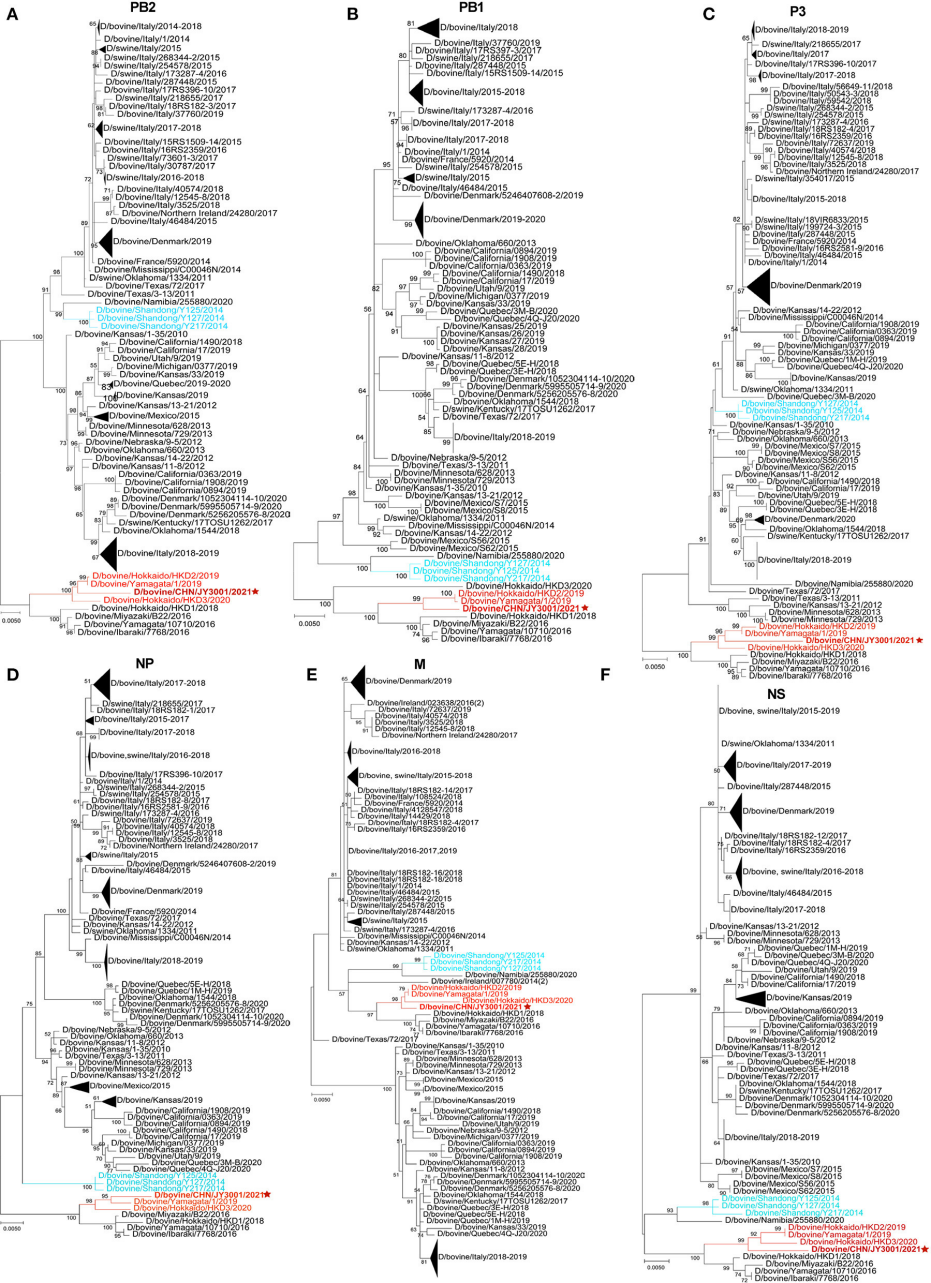
Phylogenetic trees for non-HEF segments of IDVs. **(A–F)** In MEGA-X, maximum-likelihood analysis in combination with 1,000 bootstrap replicates was used to generate trees based on the nucleotide sequences of the PB2, PB1, P3, NP, M, and NS segments. Bootstrap scores of at least 50 were shown to the left of the major nodes. Scale bar represents the number of substitutions per site. The branch of D/bovine/CHN/JY3001/2021 was in red color and marked with a star. D/Yama2019 lineage-like and D/China sub lineage-like IDV strains were labeled with red and light blue colors, respectively. The D/bovine/CHN/JY3001/2021 was bolded. Triangles indicate compressed branches with virus strains isolated in the same locations.

To further elucidate the genetic relationship of this new isolate to previously characterized IDVs, all seven genomic segments were individually analyzed by conducting phylogenetic analysis using the maximum-likelihood method in MEGA-X (12), together with IDV genome sequences retrieved from GenBank database (accessed on April 24, 2022). The HEF segment of D/bovine/CHN/JY3001/2021 clustered within the D/Yama2019 lineage (Figure 1D). The remaining genomic segments (PB2, PB1, P3, NP, M and NS) of D/bovine/CHN/JY3001/2021 were also phylogenetically close to IDV strains of D/Yama2019 lineage (Figures 2A–F). These results suggested that IDV strain within D/Yama2019 lineage is circulating in cattle population in China.

## Discussion

To date, all known IDVs identified in China cluster together and form a distinct sub-clade belonging to the D/OK lineage (9–11). However, genetic diversity of IDV remains unclear in Chinese herds. Here, we performed the genetic screening for IDV from apparently healthy cattle in slaughterhouses housing farm animals from all over the country. Of the tested nasal swab samples, IDV was detected in 10 out of 250 clinical samples (Figure 1A). Our results demonstrate that IDV continues to circulate in cattle herds in China.

The HEF segment is primarily used for phylogenetic analysis of IDV, which is the most variable gene segment of the IDV genome. Comparison of the HEF sequences of this emerging Chinese isolate and representatives of IDV lineages/sub-lineage (D/OK-, D/660-, D/Yama2016-, D/Yama2019-, D/CA2019-lineage and D/China sub-lineage) suggested that D/bovine/CHN/JY3001/2021 is a D/Yama2019 lineage-like IDV (Figures 1C,D). Corroborating this result, nucleotide sequences of all other segments of D/bovine/CHN/JY3001/2021 are close to that of D/Yama2019 lineage-like IDV strains in the phylogenetic analysis (Figure 2). To our knowledge, D/Yama2019 lineage-like IDVs have been isolated only in Japan (6, 13). This study reported the first detection of D/Yama2019 lineage-like IDV outside Japan. Therefore, further epidemiologic investigations are needed to determine the prevalence status of D/Yama2019 lineage-like IDVs.

There is no evidence of reassortments among the Chinese IDV isolates because the viruses displayed similar clustering in all phylogenetic trees according to individual genomic segments (Figures 1D, 2). Limited studies of IDVs in China combining with the small amount of Chinese IDV strains could also be the reason that we did not find evident reassortments concerning this new strain reported. Reassortment events have already been evidenced between two IDV lineages (5, 7, 14). Therefore, emergence of the D/Yama2019 lineage-like IDV in Chinese cattle herds highlights the importance of systematic virological and serological screenings to elucidate the diversity and evolution of IDVs in China.

## Data Availability Statement

The datasets presented in this study can be found in online repositories. The names of the repository/repositories and accession number(s) can be found below: https://www.ncbi.nlm.nih.gov/genbank/, ON415263-ON415269.

## Ethics Statement

Ethical review and approval was not required for this investigation because the samples used in this study were taken from cattle by veterinarians.

## Author Contributions

ML, S-LZ, and W-KW: conceptualization, supervision, and project administration. JY, TL, and ZWe: methodology and formal analysis. JY and ZWe: software, data curation, and visualization. JY, TL, S-LZ, and W-KW: validation. JY and TL: investigation. TL, SW, ZWa, JZ, MC, and FC: resources. JY: writing—original draft preparation. ML, S-LZ, W-KW, TL, and ZWe: writing—review and editing. ML, S-LZ, and JY: funding acquisition. All authors have read and agreed to the published version of the manuscript.

## Funding

This study was supported by the following grants: the Collaborative Innovation Center Fund (Nos. XTXM202202 and XT202208) and the special funds (Nos. R2020PY-JC001, R2021YJ-QG008, and 202110TD) for scientific innovation strategy-construction of high level Academy of Agriculture Science and Talent Introduction Program from Guangdong Academy of Agricultural Sciences, the grant (No. 2021A1515010524) from the Guangdong Basic and Applied Basic Research Foundation, the grant (No. 2021TDQD002) from Maoming Branch Center of Guangdong Laboratory for Lingnan Modern Agricultural Science and Technology, and the grants (Nos. 2021KJ114 and 2021KJ119) from the Department of Agriculture and Rural Affairs of Guangdong Province.

## Conflict of Interest

The authors declare that the research was conducted in the absence of any commercial or financial relationships that could be construed as a potential conflict of interest.

## Publisher's Note

All claims expressed in this article are solely those of the authors and do not necessarily represent those of their affiliated organizations, or those of the publisher, the editors and the reviewers. Any product that may be evaluated in this article, or claim that may be made by its manufacturer, is not guaranteed or endorsed by the publisher.

## Supplementary Material

Supplementary Figure S1 Sequence aligment of partial P42 gene amplicons of IDV-positive samples. The partial P42 gene sequences for IDV-positive samples ZS29-31, ZX56-58, ZS118-120, ZS130-132, ZS133-135, ZS16-18, and ZS63-65 were obtained by Sanger sequencing, and aligned together with the corresponding sequences of representative strains of the D/OK lineage, D/660 lineage, D/Yama2016 lineage, D/Yama2019 lineage, D/CA2019 lineage and D/China sub-lineage. The partial P42 gene sequences for IDV-positive samples ZS29-31, ZS130-132, ZS133-135, and ZS16-18 are identical to the corresponding sequence of D/swine/Oklahoma/1334/2011, the partial P42 gene sequences for IDV-positive samples ZX56-58 and ZS63-65 are close to the corresponding sequence of D/swine/Oklahoma/1334/2011, and the partial P42 gene sequence for the IDV-positive sample ZS118-120 is close to the corresponding sequence of D/bovine/Yamagata/1/2019.
