# Supplementary figures and images for: Identification of D/Yama2019 Lineage-Like Influenza D Virus in Chinese Cattle
Source: Front Vet Sci. 2022 Jul 14;9:939456. doi: 10.3389/fvets.2022.939456 (PMC9330358; doi:10.3389/fvets.2022.939456)

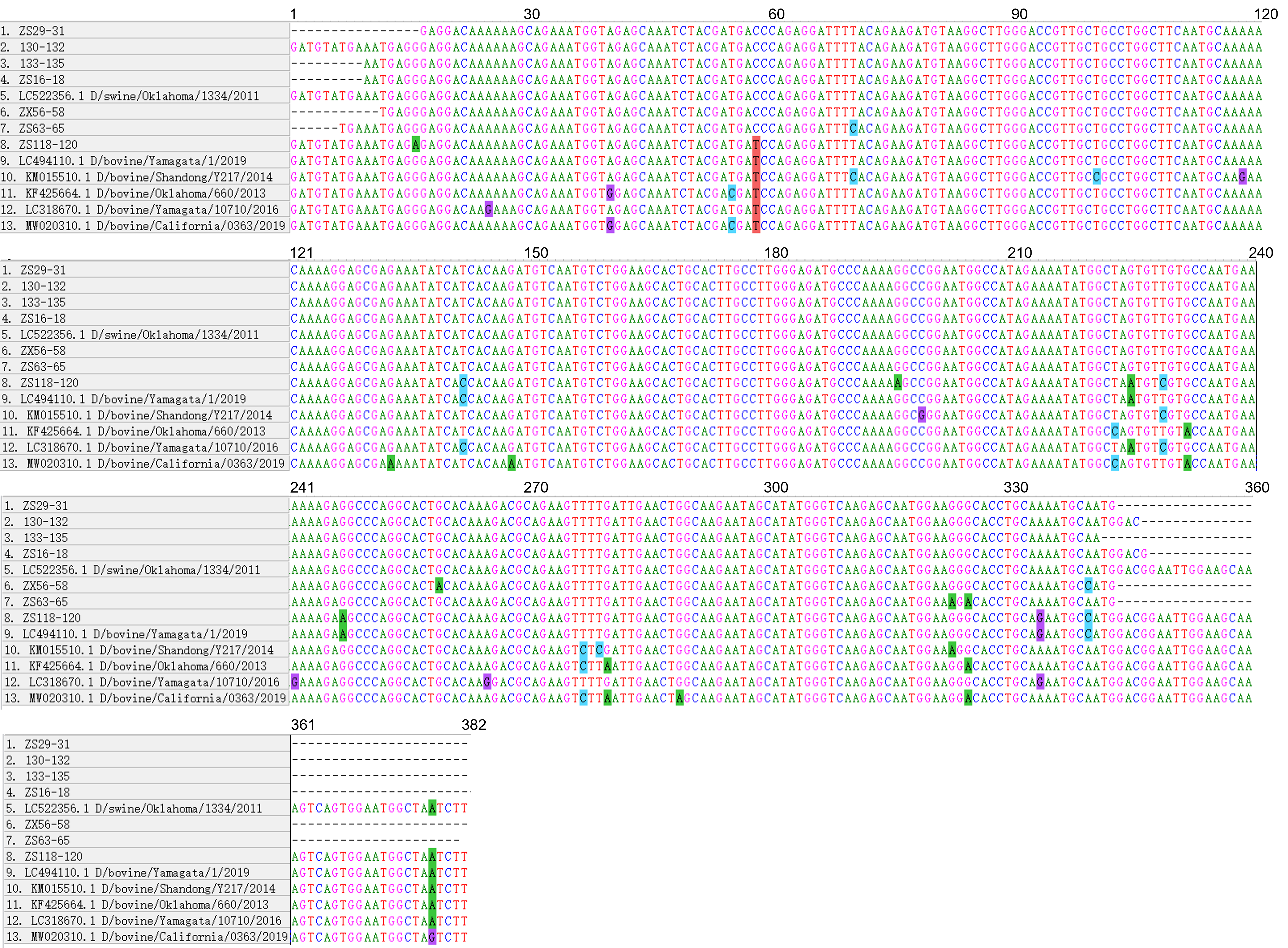

Supplement: Supplementary Figure S1 — Sequence aligment of partial P42 gene amplicons of IDV-positive samples. The partial P42 gene sequences for IDV-positive samples ZS29-31, ZX56-58, ZS118-120, ZS130-132, ZS133-135, ZS16-18, and ZS63-65 were obtained by Sanger sequencing, and aligned together with the corresponding sequences of representative strains of the D/OK lineage, D/660 lineage, D/Yama2016 lineage, D/Yama2019 lineage, D/CA2019 lineage and D/China sub-lineage. The partial P42 gene sequences for IDV-positive samples ZS29-31, ZS130-132, ZS133-135, and ZS16-18 are identical to the corresponding sequence of D/swine/Oklahoma/1334/2011, the partial P42 gene sequences for IDV-positive samples ZX56-58 and ZS63-65 are close to the corresponding sequence of D/swine/Oklahoma/1334/2011, and the partial P42 gene sequence for the IDV-positive sample ZS118-120 is close to the corresponding sequence of D/bovine/Yamagata/1/2019. [file Image_1.TIF]
